# Illicit Drug Use a Risk Factor for Ileal Mucormycosis Presenting With Acute Abdomen

**DOI:** 10.7759/cureus.12213

**Published:** 2020-12-22

**Authors:** Amaresh Aruni, Cherring Tandup, Anish Chowdhury, Arindam Roy, Bhairavi Jha

**Affiliations:** 1 Department of General Surgery, Post Graduate Institute of Medical Education and Research, Chandigarh, IND

**Keywords:** ileum, mucormycosis, perforation

## Abstract

Mucormycosis is a rare, life-threatening, angioinvasive opportunistic fungal infection. Most common sites involved are rhino cerebral, pulmonary, cutaneous followed by gastrointestinal. We report a successful management of rare gastrointestinal ileal mucormycosis with perforation peritonitis managed surgically. Later histopathology revealed the ileal mucormycosis with transmural necrotizing inflammation. Histopathological demonstration of the fungus in surgical specimens remains cornerstone of the diagnosis of mucormycosis in view of its non-specific symptoms, low isolation rates of mycological culture and lack of other rapid tests.

## Introduction

Mucormycosis is an uncommon angioinvasive fungal infection caused by the Mucorales predominantly in immunocompromised and intravenous drug addicts, and carries a poor prognosis [1]. Rhino-cerebral and pulmonary systems are most commonly involved, followed by gastrointestinal tract (GIT). In GIT, stomach, ileum and colon are the most commonly involved site in gastrointestinal system [1]. The incidence of gastrointestinal mucormycosis is increasing. We report a rare case of ileal mucormycosis managed successfully with surgery.

## Case presentation

**Figure 1:**
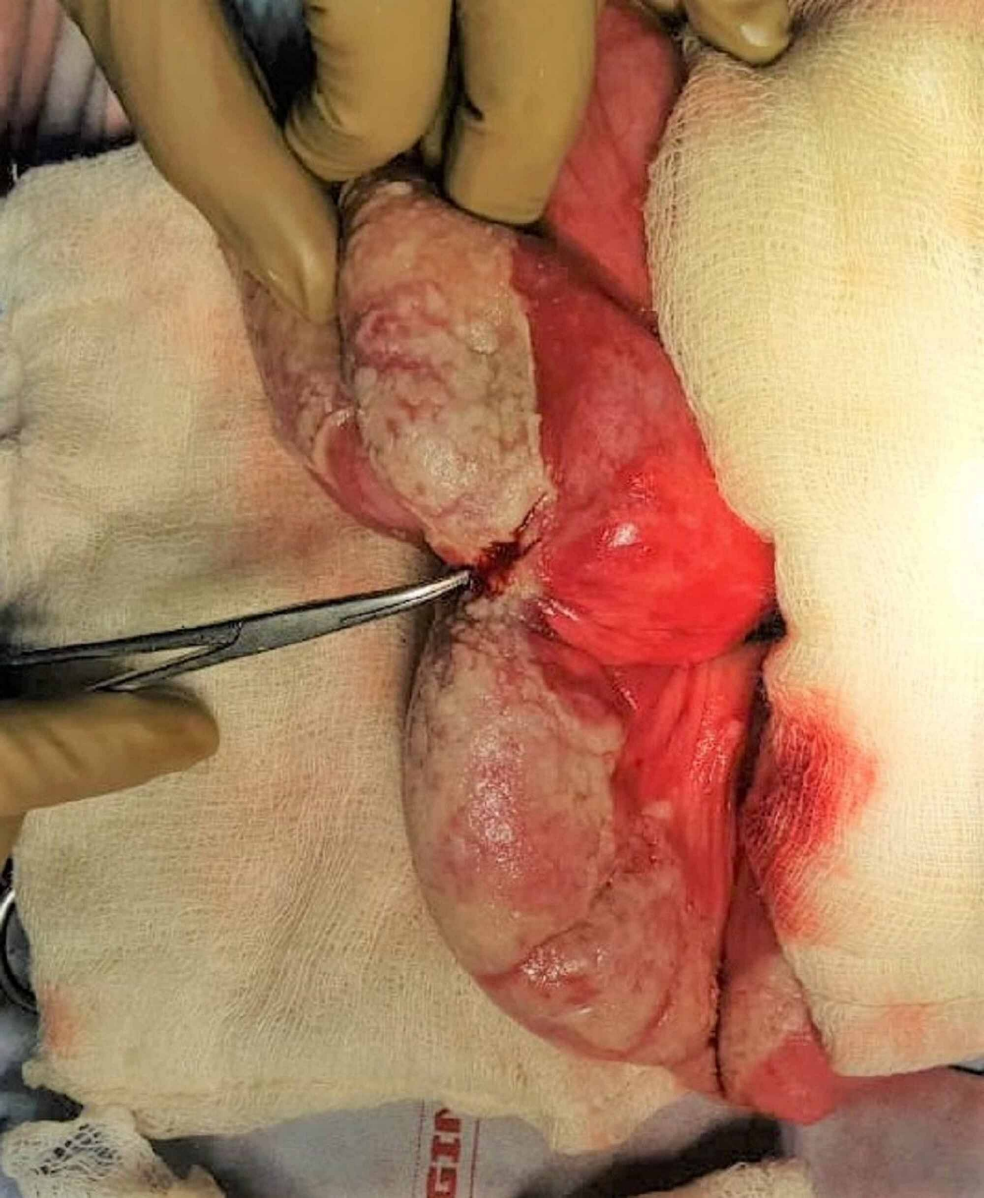
Ileal loops studded with pus flakes and perforation of size 1 x 1 cm at the stricture site.

**Figure 2:**
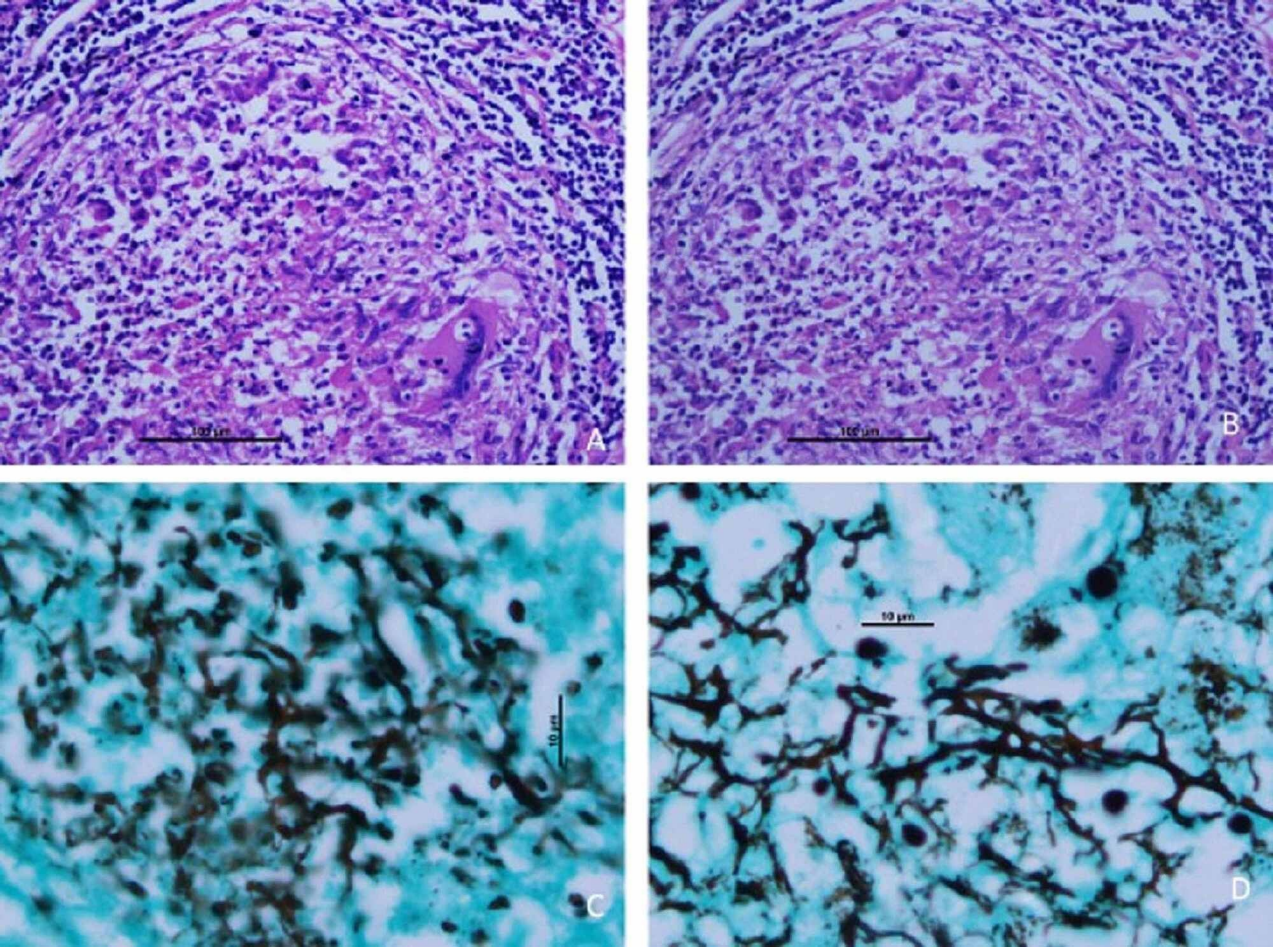
Panel of photomicrographs showing (A) and (B) are of hematoxylin eosin stained sections showing suppurative type of granulomas with many negatively stained vacuolated structures (H&E, x500). (C) and (D) are of silver staining highlighting the many easily foldable broad, aseptate right-angled branched fungal profiles present in the suppurative foci and also within the multinucleated giant cells (methenamine silver nitrate, x1000).

A 49-year-old male addicted to intravenous drug abuse, presented with severe pain abdomen, abdomen distension, constipation and bilious vomiting for four days. On examination, abdomen revealed a peritonitis sign and was having tachypnea and tachycardia and blood pressure was 90/60 mmHg. Blood parameters showed leucocytosis with TLC- 24,000 cubic millimeters, ABG showed mild metabolic acidosis pH being 7.23, and renal and liver profile was normal, blood culture was sterile. Anti-hepatitis C virus (HCV) enzyme-linked immunosorbent assay (ELISA) was positive. Erect X-ray of the abdomen showed air under the diaphragm. He was resuscitated and with the diagnosis of perforation peritonitis underwent exploratory laparotomy, intra-operative findings revealed around 1 litre biliopurulent free fluid in the peritoneum along with ileal perforation of size approx. 1 x 1 cm which was proximal to the stricture segment of the ileal loop (Figure. 1), multiple pus flakes were present over the bowel. We did resection of the perforated ileal segment and did double barrel ileostomy. Postoperatively patient did well, oral intake was started by day 2, and was discharged by day 6. On follow-up, anti-HCV drugs were started due to raised HCV-RNA viral load. He also underwent deaddiction therapy for intravenous addiction. Surprisingly histopathology report came out as transmural necrotizing inflammation with multiple suppurative granulomas. These granulomas showed many negative shadows (Figure 2A and B) in hematoxylin eosin-stained sections. Methenamine silver nitrate staining was carried out to identify the negative shadows and showed many easily foldable friable fungal profiles with right-angled branching (Figure 2C and D) conforming to the morphology of mucormycosis. He did not receive anti-fungal medication as he was discharged earlier than the pathology report and was doing well in follow up. At five-month follow-up on anti-HCV therapy with no intravenous addiction and planned for restoration of bowel continuity.

## Discussion

Mucormycosis is an angioinvasive, life-threatening opportunistic fungal infection caused by fungi of the subphylum Mucormycotina, order Mucorales [2]. Rhizopus species are the organisms that most commonly cause mucormycosis in humans (34%-47%), followed by Mucor (18%-19 %) [3]. Other less frequently isolated species include Rhizomucor, Lichtheimia, Cunninghamella, Saksenaea, and Apophysomyces [4]. Mucorales are ubiquitous in nature, often being found in decaying organic matter [4]. Mucormycosis was first reported by Paultauf in 1885 as a cause of human disease [4]. The incidence of mucormycosis is approximately 1.7 cases per 1,000,000 inhabitants per year in the USA [5]. In a large study of more than 900 reported cases, the major involved sites are rhino-cerebral (21%), pulmonary (24%), cutaneous (19%), localized cerebral (9%), and gastrointestinal (7%) [4]. Any part of the gastrointestinal tract can be involved, stomach is the most common site (57.5%), followed by colon (32.2%), small intestine is less commonly involved (10.3%); jejunum is the least likely site (1.1%) [5]. In small bowel, ileocecal region is the most common site involved this may challenge in differentiating from typhilitis both clinically and radiologically [6]. GI mucormycosis is more common in children (70%) than in adults. Neonatal GI mucormycosis is a unique; pre-term neonates are at high risk, colon is typically involved and clinical presentation resembles necrotizing enterocolitis [3].

Risk factors for mucormycosis are neutropenia, immunosuppression, diabetes, penetrating trauma, prematurity, malnourishment, hematologic malignancies, burns, iron overload and illicit intravenous drug use as in our case [1]. HIV infection is not a risk factor for mucormycosis; neutrophils rather than lymphocytes are crucial for defence. Notably, 19% to 54% of patients with mucormycosis do not have any identifiable risk factor [3]. The major modes of transmission for human Mucorales infection include inhalation, ingestion and cutaneous exposure. In our case, small bowel involvement without any other organ strongly suggests a gastrointestinal portal of entry [1]. It is acquired by ingestion of pathogens in foods such as fermented milk, dried bread products and fermented porridge, also by contaminated wooden tongue depressors. Other sources include contaminated intravenous fluids, adhesive tapes, ostomy bags, catheters, drains, peritoneal dialysis and intravascular devices [7]. The symptoms of gastrointestinal mucormycosis are non-specific pain abdomen, abdomen distension, nausea, vomiting, fever, haematochezia or perforation leading to peritonitis [2,4]. Mucormycosis is angioinvasive leading to bowel ischemia, necrosis, perforation, peritonitis or massive hemorrhage in gastrointestinal system [8]. The diagnosis is often delayed due to non-specific presentation and only 25% of cases are diagnosed ante mortemand has high mortality rate up to 85% hence requires high index of suspicious and early biopsy [1,4]. Histopathologic examination of tissues with Mucorales infection typically shows characteristic broad, ribbon-like hyphae with a few or no septa and wide-angled branching, accompanied by tissue necrosis and angioinvasion which is confirmatory and excludes aspergillosis [1,3]. Culture isolation ranges 50%-71% in autopsy cases and 30% in surgical specimens [9], hence histopathologic diagnosis remains cornerstone of invasive mucormycosis [3]. Mucorales-specific real-time polymerase chain reaction assay identifies DNA of Mucorales species and confirms diagnosis. Fungal markers such as ß-D-glucan and galactomannan tests do not detect the antigenic components of the Mucorales cell wall [1,4]. The mainstay of treatment of mucormycosis is early diagnosis, antifungal therapy with liposomal Amphotericin-B(LAmB), surgical debridement and reversal of the risk factors. Aggressive surgical debridement is of utmost importance because thrombosis and tissue necrosis prevent the penetration of the antifungal agents to the site of infection. In our case, we resected the entire diseased ileal segment and the omentum, which helped in the successful recovery of the patient despite delayed diagnosis by histopathology and no antifungal therapy. Liposomal Amphotericin-B has a long mean residence time in tissues up to two weeks after cessation of the drug. Posoconazole is the second-line or salvage therapy in cases refractory to LAmB [1].

## Conclusions

Gastrointestinal mucormycosis is a rare angioinvasive fungal infection associated with high mortality. High index of suspicious should be kept in cases of immunosuppressed and intravenous drug addiction patients with acute abdomen, early diagnosis and surgical therapy is of utmost importance. LAmB is the drug of choice.
